# Se “Tempo é Músculo”, então os Conhecimentos do Paciente devem Economizar Tempo

**DOI:** 10.36660/abc.20220392

**Published:** 2022-07-07

**Authors:** Daniel Ferreira

**Affiliations:** 1 Hospital da Luz Digital Lisboa Portugal Hospital da Luz Digital, Lisboa – Portugal; 2 Serviço de Medicina Intensiva Hospital da Luz Lisboa Lisboa Portugal Serviço de Medicina Intensiva – Hospital da Luz Lisboa, Lisboa – Portugal

**Keywords:** Infarto do Miocárdio, Intervenção Coronária Percutânea/métodos, Conscientização, Reperfusão Miocárdica, Assistência Integral à Saúde/economia, Isquemia Miocárdica/terapia

Há mais de meio século, o trabalho experimental do grupo de Eugene Braunwald sobre os fatores que influênciam o tamanho do infarto após oclusões das artérias coronárias levou ao conceito de “Tempo é músculo” no que se refere ao manejo do infarto agudo do miocárdio.^1^

A reperfusão atempada das artérias coronárias ocluídas é fundamental para salvar as células miocárdicas isquêmicas em risco no infarto agudo do miocárdio com elevação do segmento ST (STEMI).

Nas últimas décadas, o foco tem sido colocado nos esforços para encurtar os tempos porta-agulha ou porta-balão e buscar melhores e mais seguras modalidades de terapias de reperfusão.

Quando diferentes modalidades de reperfusão devem ser consideradas, a duração dos sintomas e o tempo esperado para atingir a reperfusão são fundamentais para a escolha da melhor terapia para cada paciente. Esse conceito levou à comparação da terapia farmacológica lítica, iniciada na fase pré-hospitalar ou em hospitais sem laboratório de cateterismo, e intervenção coronária percutânea – ICP.^2^

Independentemente da estratégia de reperfusão escolhida (lítica ou ICP), o tempo desde o início dos sintomas até a reperfusão bem-sucedida é fundamental para o prognóstico dos pacientes a curto e longo prazo.^3,4^

Para citar o artigo histórico de Elliott M. Antman: “*No futuro, os avanços no cuidado de pacientes com infarto do miocárdio com supradesnivelamento do segmento ST (IAMCST) não virão da análise de estudos que não refletem a prática atual em um esforço para racionalizar o prolongamento do tempo de atraso relacionado à intervenção coronária percutânea (ICP). Devemos ir além desses argumentos e encontrar maneiras de encurtar o tempo total de isquemia*.”^5^

Terkelsen et al.,^6^ dividiram o tempo total de isquemia em ‘atraso do paciente’ e ‘atraso do sistema’, sugerindo que o último, mas não o primeiro, pode ser influenciado pelo profissional de saúde.

As diretrizes STEMI da Sociedade Europeia de Cardiologia de 2017^7^ indicam que todos os componentes do atraso do sistema (determinado como o intervalo do primeiro contato médico (PCM) até a reperfusão) representam a qualidade do atendimento, sendo recomendado mensurá-los como indicadores de qualidade.

No entanto, como mencionado acima, o tempo isquêmico total é o principal determinante do tamanho do infarto no STEMI. A ênfase foi colocada na redução do componente de tempo de terapia porta-reperfusão (o chamado atraso do sistema), enquanto o sintoma-a-PCM (o atraso do paciente) é muitas vezes esquecido.

O atraso do paciente pode ser atribuído a várias características individuais, mas também sociais dos pacientes que apresentam STEMI. Vários artigos abordaram essa questão e descobriram que a decisão de procurar ajuda médica, ligando para os serviços de emergência ou apresentando-se a um centro médico, pode variar de pessoa para pessoa. No entanto, foram identificadas algumas características comuns que justificam a apresentação tardia dos pacientes ao primeiro contato médico.^8–11^

Neste número dos Arquivos Brasileiros de Cardiologia, Khalfallah et al.,^12^ apresentam uma avaliação muito interessante de dois fatores que influenciam o atraso do paciente na reperfusão por ICP.^12^

A conscientização do paciente sobre os sintomas relacionados à isquemia miocárdica e que esses sintomas podem alertar para uma doença grave (até mesmo com risco de vida) é um importante determinante da decisão atempada de procurar atendimento médico. Campanhas direcionadas para aumentar a conscientização do paciente têm mostrado resultados mistos, principalmente devido a diferentes abordagens que buscam melhorar a educação em saúde das populações em risco.^8,12^

Outro aspecto relevante da conscientização do paciente é o conhecimento do paciente sobre os benefícios da reperfusão precoce. Khalfallah et al.,^12^ verificaram que a conscientização dos pacientes sobre os benefícios da revascularização precoce foi significativamente menor em pacientes com apresentação tardia, o que eles sugerem que pode ser outro motivo para a procura tardia de aconselhamento médico.^12^

O outro achado relevante deste trabalho é a relação entre os fatores socioeconômicos dos pacientes e o momento da apresentação do paciente ao atendimento médico. Os autores realizaram uma análise de regressão multivariada para identificar os preditores socioeconômicos independentes que afetam a apresentação do paciente à ICP e descobriram que a proporção de pacientes com baixa escolaridade foi significativamente maior no grupo de apresentação tardia.^13^ Além disso, os pacientes que sofriam de isolamento social e os que moravam sozinhos foram mais prevalentes nesse grupo. Como os autores discutem, esses achados estão de acordo com outros estudos sobre o tema,^14,15^ mas esta é outra área de relatos conflitantes, com outros autores relatando nenhuma relação entre fatores socioeconômicos e tempo de apresentação.^13^

Podemos, assim, concluir que esta é uma temática de grande interesse e investigação em curso e que são bem-vindos mais estudos que procurem avaliar o impacto da educação em saúde no prognóstico de doentes com STEMI. No entanto, as evidências mostram que os profissionais de saúde devem continuar prestando o melhor atendimento possível (incluindo reperfusão oportuna) aos apresentadores precoces e tardios.^16^

Os profissionais de saúde, principalmente os responsáveis pelo atendimento de pacientes de alto risco, devem aproveitar qualquer oportunidade para melhorar a educação em saúde de seus pacientes em relação aos sintomas relacionados à isquemia miocárdica, os riscos de apresentação tardia ao atendimento médico e os benefícios da reperfusão precoce no caso de suspeita de infarto do miocárdio.
