# Supplementary material for: Se “Tempo é Músculo”, então os Conhecimentos do Paciente devem Economizar Tempo
Source: Arq Bras Cardiol. 2022 Jul 7;119(1):35–6. [Article in Portuguese] doi: 10.36660/abc.20220392 (PMC9352136; doi:10.36660/abc.20220392)
Supplement: Translation

# Translation: If “Time Is Muscle,” Then the Patient's Knowledge Must Save Time

**Authors:** Daniel Ferreira

**Affiliations:** 1 Lisboa Portugal Hospital da Luz Digital, Lisboa – Portugal; 2 Lisboa Portugal Serviço de Medicina Intensiva – Hospital da Luz Lisboa, Lisboa – Portugal

**Keywords:** Myocardial Infarction, Percutaneous Coronary Intervention/methods, Awareness, Myocardial Reperfusion, Comprehensive Health Care/economics, Myocardial Ischemia/therapy

More than half a century ago, Eugene Braunwald's group experimental work on the factors influencing infarct size following coronary artery occlusions led to the concept of “Time is Muscle” in what relates to acute myocardial infarction management.^1^

Timely reperfusion of the occluded coronary arteries is critical to saving at-risk ischemic myocardial cells in acute ST-elevation myocardial infarction (STEMI).

In the last decades, the focus has been put on the efforts to shorten the door-to-needle or door-to-balloon times and look for better and safer modalities of reperfusion therapies.

When different reperfusion modalities are to be considered, the duration of symptoms and the expected time to reach reperfusion are key to choosing the best therapy for each patient. This concept has led to the comparison of lytic pharmacologic therapy, initiated in the pre-hospital phase or at hospitals without cath lab facilities, and percutaneous coronary intervention – PCI.^2^

Independently of what reperfusion strategy is chosen (lytics or PCI), the time from symptom onset to successful reperfusion is critical to the short- and long-term patients’ prognosis.^3,4^

To quote Elliott M. Antman's landmark paper: “*In the future, advances in the care of patients with ST-segment elevation myocardial infarction (STEMI) will not come from the analysis of trials that do not reflect current practice in an effort to rationalize extending the percutaneous coronary intervention (PCI)-related delay time. We must move beyond such arguments and find ways to shorten the total ischemic time.*”^5^

Terkelsen et al. ^6^ divided the total ischaemic time into ‘patient delay’ and ‘system delay,’ suggesting that the latter, but not the former, can be influenced by the healthcare provider.

The 2017 European Society of Cardiology STEMI guidelines^7^ indicates that all components of the system delay (determined as the interval from first medical contact (FMC) to reperfusion) represent the quality of care, and it is recommended to measure them as quality indicators.

Nevertheless, as mentioned above, total ischemic time is the major determinant of infarct size in STEMI. Emphasis has been placed on reducing the door-to-reperfusion therapy time component (the so-called system delay), whereas the symptom-to-FMC (the patient delay) is often overlooked.

Patient delay can be attributed to several individuals and societal characteristics of the patients presenting with STEMI. Several papers have addressed this issue and found that the decision to seek medical help by calling the emergency services or self-presenting to a medical facility can vary from person to person. However, some common characteristics have been identified that justify the late presentation of patients to first medical contact.^8–11^

In this issue of Arquivos Brasileiros de Cardiologia, Khalfallah et al.^12^ present a very interesting evaluation of two factors that influence patient delay in PCI reperfusion.^12^

Patient awareness of myocardial ischemia-related symptoms and that those symptoms might alert to a serious (even life-threatening) disease is a major determinant of the timely decision to seek medical care. Campaigns directed to increase patient awareness have shown mixed results, mostly due to different approaches seeking to improve the health literacy of the at-risk populations.^8,12^

Another relevant aspect of patient awareness is the patient's knowledge of early reperfusion benefits. Khalfallah et al.^12^ found that awareness of the patients about the benefits of early revascularization was significantly lower in late presenting patients, which they suggest might be another reason for late seeking medical advice.^12^

The other relevant finding of this paper is the relationship between patients’ socioeconomic factors and the timing of patient presentation to medical care. The authors performed a multivariate regression analysis to identify the independent socioeconomic predictors affecting patient presentation to PCI and found that the proportion of patients with low educational levels was significantly higher in the late presenting group.^13^ Also, patients who suffered from social isolation and those that lived alone were more prevalent in this group. As the authors discuss, these findings are in line with other studies on this topic,^14,15^ but this is another area of conflicting reports, with other authors reporting no relationship between socioeconomic factors and timing of presentation.^13^

We can thus conclude that this is a matter of great interest and ongoing research and that more studies seeking to evaluate the impact of health literacy on the prognosis of STEMI patients are welcome. Nevertheless, evidence shows that healthcare providers must continue giving the best care possible (including timely reperfusion) to early and late presenters.^16^

Healthcare professionals, particularly those responsible for caring for high-risk patients, must grab any opportunity to improve their patients’ health literacy concerning myocardial ischemia-related symptoms, the risks of later presentation to medical care, and the benefits of early reperfusion in the case of suspected myocardial infarction.
